# Resectable adenocarcinoma developing in the remnant pancreas 7 years after partial pancreatoduodenectomy for invasive ductal adenocarcinoma of the pancreas: a case report

**DOI:** 10.1186/s13256-017-1359-3

**Published:** 2017-07-17

**Authors:** Lina Frei, Ruedi Stieger, Christian Bayerl, Stefan Breitenstein, Ralph F. Staerkle

**Affiliations:** 1 Clinic for Visceral and Thoracic Surgery, Kantonsspital Winterthur, Brauerstrasse 15, 8401 Winterthur, Switzerland; 2 Institute for Pathology, Kantonsspital Winterthur, Brauerstrasse 15, 8401 Winterthur, Switzerland

**Keywords:** Recurrent pancreatic adenocarcinoma, Repeat pancreatectomy, Long-term survivors

## Abstract

**Background:**

Pancreatic adenocarcinoma still has an excessively high mortality rate and resection is the only potentially curative treatment. The postoperative 5-year survival rate is approximately 20% and recurrence develops generally within 2 years. We report a case of a localized recurrent pancreatic adenocarcinoma in the remnant pancreas, 7 years after initial resection.

**Case presentation:**

In 2008 an abdominal computed tomography scan showed a mass in the pancreas of a 70-year-old white woman, who presented with occlusive jaundice and abdominal pain in her right upper quadrant. A pancreatoduodenectomy was performed for a clinically suspected pancreatic adenocarcinoma. Histology confirmed a ductal adenocarcinoma. Afterwards, she received adjuvant chemotherapy with gemcitabine. In 2015 a follow-up positron emission tomography-computed tomography scan showed a single lesion in her remnant pancreas. Subsequently, a partial re-resection was performed. Histology confirmed once again a ductal adenocarcinoma. She received adjuvant chemotherapy with gemcitabine again and is still alive almost 9 years after she had first been diagnosed as having pancreatic cancer.

**Conclusions:**

In selected cases re-resection for pancreatic recurrence is feasible and provides a survival benefit. In cases involving late recurrence, it is difficult to distinguish between true recurrence and the development of a new tumor. In order to detect recurrences at an early stage in long-term survivors, follow-up needs to occur on a regular and long-term basis.

## Background

Out of all pancreatic cancers, 80% are pancreatic ductal adenocarcinomas. The worldwide incidence is 4.9 per 100,000 annually for men and 3.6 per 100,000 annually for women [1, 2]. Due to its aggressive nature, surgery is generally the only option for curative treatment. But due to locally advanced infiltration or local and systemic metastases, only a third of the patients are eligible for surgery at initial diagnosis [3].

The last few decades have seen major advancements in surgical techniques and adjuvant therapy options for the management of pancreatic malignancies. Perioperative mortality has been significantly reduced to less than 5%. However, the overall 5-year survival rate after resection remains low with reported rates of 22.5% in combination with adjuvant chemotherapy and 11.5% without adjuvant chemotherapy [3, 4]. This reflects the high mortality rate of 4.7 per 100,000 annually for men and 3.4 per 100,000 annually for women [2].

Approximately 80% of all surgically treated patients develop a recurrence [5]. Usually the recurrence is found in the remnant pancreas or has metastasized to the liver or the peritoneum. It typically develops within 2 years after the initial resection [6].

It is therefore exceptional that we can report the case of a woman with successful re-resection of recurrent pancreatic ductal adenocarcinoma in the remnant pancreas, 7 years after initial resection.

## Case presentation

**Fig. 1:**
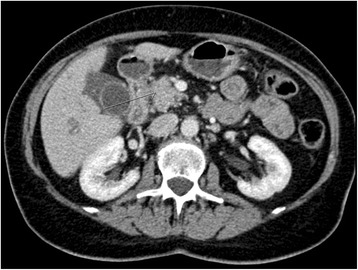
August 2008. Contrast-enhanced abdominal computed tomography shows a tumor in the head/uncinate process of the pancreas

In 2008, a 70-year-old white woman with a medical history of hypertension, dyslipidemia, and gonarthrosis was referred to a regional hospital by her general practitioner with occlusive jaundice and abdominal pain in her right upper quadrant. An abdominal computed tomography (CT; Fig. 1) scan showed a 2 × 1.7 cm mass in the head and uncinate process of her pancreas with abutment of her portal vein. Her cancer antigen 19-9 (CA 19-9) was 12.3 kU/l.

**Fig. 2:**
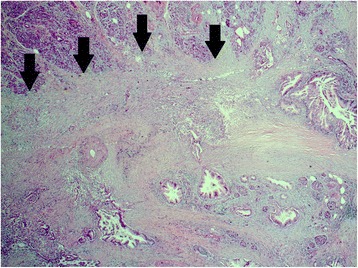
September 2008. Histological specimen with hematoxylin-eosin staining; magnification × 25. *Arrows* indicate the area of the carcinoma

Although repeated fine needle aspiration failed to confirm the radiologically suspected carcinoma, it remained the leading differential diagnosis. The case was discussed at a multidisciplinary tumor conference; in accordance with the conference a pancreatoduodenectomy (PD) with portal vein resection was performed in September 2008. Histopathology (Fig. 2) showed a ductal adenocarcinoma: pT3, pN1 (1/31), G3, V1, R0. Her postoperative course was uneventful and she received a full course of adjuvant chemotherapy with six cycles of gemcitabine from October 2008 to April 2009.

**Fig. 3:**
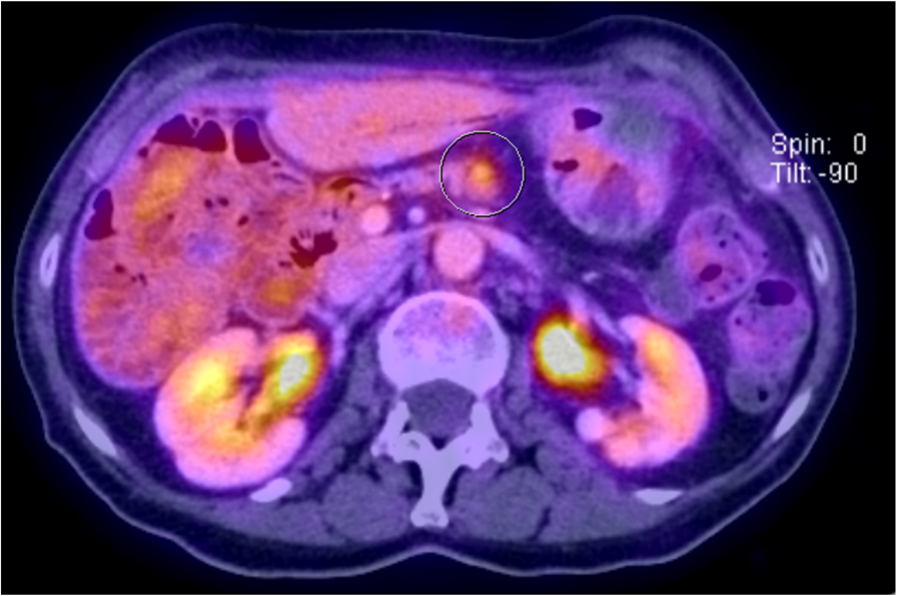
August 2015. Abdominal positron emission tomography-computed tomography shows the tumor in the pancreatic remnant

For the first 4.5 years (54 months) clinical follow-ups were carried out every 3 months, and controls of CA 19-9 levels as well as radiological controls were performed every 6 months. Subsequently, radiological controls and controls of CA 19-9 levels were performed annually. From 2012 onwards, our patient complained of recurrent fever. As CT scans showed no evidence of recurrence, a positron emission tomography (PET)-CT was performed in August 2015 (Fig. 3). It showed a single suspicious lesion in the body of her remnant pancreas, 3 cm distal of the pancreaticojejunostomy without any evidence for distant metastasis or peritoneal dissemination. At this point her CA 19-9 was in normal range at 5.0 kU/l.

**Fig. 4:**
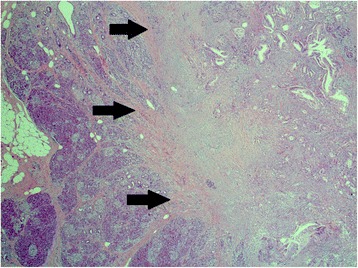
September 2015. Histological specimen with hematoxylin-eosin staining; magnification × 25. *Arrows* indicate the area of the carcinoma

Her case was once again discussed at a multidisciplinary tumor conference, and an indication for re-resection was confirmed. In September 2015 a resection of the tumor in her remnant pancreas was performed. Intraoperatively it seemed possible to achieve clear resection margins while preserving the tail of her pancreas. In addition, the diameter of her pancreatic duct (3 mm) seemed to be wide enough to perform a safe pancreaticojejunal anastomosis. Therefore, we refrained from performing a total pancreatectomy and decided on a new pancreaticojejunostomy with a Roux-en-Y reconstruction. A histopathological work-up (Fig. 4) showed a ductal adenocarcinoma once again: pT3, pN0 (0/6), G2, R0.

After recovery, she started another course of adjuvant chemotherapy with six cycles of gemcitabine from November 2015 to May 2016. Since May 2016 she has been under regular surveillance, with clinical follow-ups and controls of CA 19-9 levels every 3 months. A CT scan is performed biannually. The first CT in November 2016 showed no recurrence or evidence for metastasis and her CA 19-9 was within normal range at 2.7 kU/l.

She is still alive and well today, 8 years and 9 months after the first operation and 1 year and 9 months after the second operation.

**Fig. 5:**
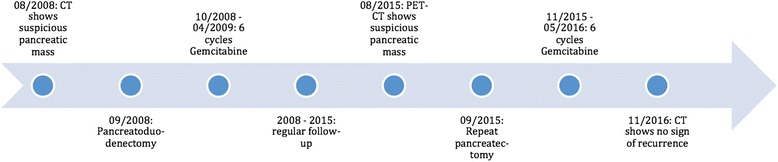
Timeline

A timeline is shown in Fig. 5.

## Discussion

Over the last 10 years only a few similar case reports have been published [7–13]. Due to the aggressive nature of pancreatic adenocarcinoma and the poor 5-year survival rate after resection, the possibility of surgical treatment of a recurrence is rare.

Miyazaki *et al.* published a retrospective study analyzing the cases of 284 patients who underwent R0 or R1 resection for pancreatic adenocarcinoma between 2000 and 2012 [14]. Of these patients, 170 developed a recurrence by April 2013. Of the 170 patients, 11 were eligible for re-resection of an isolated recurrence in the remnant pancreas. The 5-year survival rate in the repeat pancreatectomy group versus the unresectable group was 41% versus 6.2% (*P* < 0.01). Miyazaki *et al.* concluded that repeat pancreatectomy may improve the survival rate, but should only be sought if R0 resection can be accomplished [14]. Similar results were found in retrospective studies by Strobel *et al.* and Kleeff *et al.* where the median survival time in the repeat pancreatectomy group versus the unresectable group was 16.4 versus 9.4 months and 17 versus 9.4 months respectively [5, 15]. Repeat pancreatectomy seems to be a safe method for selected patients with isolated local recurrence, especially if R0 resection can be achieved. Currently there is no official guideline for the treatment of isolated recurrence in the remnant pancreas. By evaluating the CT from our patient, we concluded that a re-resection with negative margins could be achieved and was technically feasible.

As most recurrences develop within the first 2 years after resection [6], there is some disagreement about the true nature of recurrent pancreatic cancer in the remnant pancreas at a later stage [8, 9, 16].

Hashimoto *et al.* reported the cases of eight patients who underwent completion pancreatectomy for cancer in the remnant pancreas 23 to 103 months (median 35.5 months) after initially undergoing PD/distal pancreatectomy for pancreatic cancer [16]. They compared histological specimens from the initial resection with specimens from the repeat resection by means of pyrosequencing assay for Kirsten rat sarcoma (KRAS) mutations and immunohistochemistry for mucin (MUC) 1 and MUC2 to distinguish between cancer recurrence and new primary lesion. Their findings led them to suspect four cases to be true local recurrences of the initial cancer and four cases to be new primary adenocarcinomas. That being said, there is evidence that the genetic composition of a single pancreatic tumor is rather heterogenic [17]. Other authors distinguish between recurrence and new primary lesion based on the location of the recurrence, that is, whether the recurrence is “at or near the original plane of transection” and the time between the first and second cancer [8, 9, 18]. It is still difficult to be certain of a tumor’s true etiology. In our case, the patient developed the second lesion in the body of her remnant pancreas, well away from the resection margin. In addition, considering the time between the first and second cancer, we believe that she developed a new primary carcinoma rather than a recurrence.

To date, there is no scientific evidence supporting the belief that regular follow-ups, as defined by staging examinations after resection of a pancreatic cancer, have an influence on survival rate. Neither the German S3 guidelines [19] nor the European Society for Medical Oncology (ESMO) guidelines [1] support regular structured follow-ups. However, the reported level of evidence for both guidelines is very low. The advocates of these guidelines argue that in the great majority of cases the situation is palliative, if a recurrence occurs, and the overall prognosis is poor.

The US National Comprehensive Cancer Network (NCCN) guidelines recommend postoperative follow-ups after curative resection of pancreatic adenocarcinoma every 3 to 6 months for 2 years, followed by annual controls [20]. This is based on the fact that most of the recurrences develop within the first 2 years after surgery. As recurrences and/or new primary cancers can develop from 7 up to 107 months postoperatively, Ishida *et al.* recommended a more closely monitored follow-up [18]: They concluded that follow-ups should be carried out every 3 months for 10 years after surgery to detect a possible recurrence or a new lesion at an early stage. A small group of long-term survivors may benefit from a more close-meshed follow-up after pancreatic cancer surgery. Needless to say from an economic point of view such a close-meshed follow-up is difficult to support, since long-term survival is extremely rare.

In our case, clinical controls were performed every 3 months and controls of CA 19-9 as well as radiological controls every 6 months for the first 54 months, followed by annual controls. The same pattern of surveillance, with more frequent controls of CA 19-9, was chosen after the second operation.

The extent of re-resection is controversially discussed in the literature. In all of the cited case reports a completion pancreatectomy was performed [7–13]. Prior to the surgery in our reported case, we discussed the extent of the re-resection. On the one hand, partial resection would spare the patient from endocrine and exocrine pancreatic insufficiency and its comorbidities. On the other hand, partial resection would promote the risk of new recurrence in the remnant pancreas. In the end, the decision is between a better quality of life for the patient and radical oncological surgery. As reported above, our final decision was made intraoperatively. We strongly believe that if clean margins can be achieved, partial pancreatectomy is a valuable alternative to completion pancreatectomy, even in the case of a repeat resection for recurrent pancreatic adenocarcinoma. From an oncological standpoint there are no data available on the recommended extent of resection in these rare situations.

## Conclusions

Re-resections are worthwhile in selected cases and are statistically significantly beneficial for patients in terms of survival rates. Currently, there are no official guidelines for the treatment of resectable recurrent pancreatic adenocarcinoma and the decision has to be made on an individual basis, by a multidisciplinary panel.

For long-term survivors undergoing resection of pancreatic adenocarcinoma, long-term surveillance seems to be mandatory. The revision of existing guidelines appears to be inevitable, since there are considerable discrepancies regarding postoperative follow-up.

In selected patients partial pancreatectomy is technically feasible and saves patients from the lifelong endocrine and exocrine pancreatic insufficiency caused by total pancreatectomy. Currently, there are no recommendations on the extent of re-resection; therefore, we suggest that further studies be conducted on the matter.

## Abbreviations

CA 19-9: Cancer antigen 19-9
CT: Computed tomography
ESMO: European Society for Medical Oncology
KRAS: Kirsten rat sarcoma
MUC: Mucin
NCCN: National Comprehensive Cancer Network
PD: Pancreatoduodenectomy
PET: Positron emission tomography

## References

[1] Ducreux M, Cuhna AS, Caramella C, Hollebecque A, Burtin P, Goéré D, *et al.* Cancer of the pancreas: ESMO Clinical Practice Guidelines for diagnosis, treatment and follow-up. Ann Oncol. 2015;26 Suppl 5:v56–68. 10.1093/annonc/mdv295 26314780

[2] Ferlay J, Soerjomataram I, Dikshit R, Eser S, Mathers C, Rebelo M, *et al.* Cancer incidence and mortality worldwide: sources, methods and major patterns in GLOBOCAN 2012. Int J Cancer. 2015;136(5):E359–86. doi:10.1002/ijc.29210. 25220842

[3] Riall TS, Lillemoe KD. Underutilization of surgical resection in patients with localized pancreatic cancer. Ann Surg. 2007;246(2):181–2. doi:10.1097/SLA.0b013e31811eaa2c. PMC1933567 17667494

[4] Oettle H, Post S, Neuhaus P, Gellert K, Langrehr J, Ridwelski K, *et al.* Adjuvant chemotherapy with gemcitabine vs observation in patients undergoing curative-intent resection of pancreatic cancer: a randomized controlled trial. JAMA. 2007;297(3):267–77. doi:10.1001/jama.297.3.267. 17227978

[5] Strobel O, Hartwig W, Hackert T, Hinz U, Berens V, Grenacher L, *et al.* Re-resection for Isolated Local Recurrence of Pancreatic Cancer is Feasible, Safe, and Associated with Encouraging Survival. Ann Surg Oncol. 2013;20(3):964–72. doi:10.1245/s10434-012-2762-z. 23233235

[6] Sperti C, Pasquali C, Piccoli A, Pedrazzoli S. Recurrence after resection for ductal adenocarcinoma of the pancreas. World J Surg. 1997;21(2):195–200. 10.1007/s002689900215 8995078

[7] Akabori H, Shiomi H, Naka S, Murakami K, Murata S, Ishida M, *et al.* Resectable carcinoma developing in the remnant pancreas 7 years and 10 months after distal pancreatectomy for invasive ductal carcinoma of the pancreas: report of a case. World J Surg Oncol. 2014;12(1):224. doi:10.1186/1477-7819-12-224. PMC4107580 25034035

[8] Hamner JB, White M, Crowder C, Singh G. Resection of metachronous pancreatic cancer 4 years after pancreaticoduodenectomy for stage III pancreatic adenocarcinoma. World J Surg Oncol. 2015;13(1):290. doi:10.1186/s12957-015-0712-5. PMC4588897 26419361

[9] Hardacre JM. Completion Pancreaticoduodenectomy for a Second Primary Pancreatic Cancer: A Case Report. Case Rep Pancreat Cancer. 2016;2(1):50–2. doi:10.1089/crpc.2016.0010. PMC6319681 30631816

[10] Koizumi M, Sata N, Kasahara N, Morishima K. Remnant pancreatectomy for recurrent or metachronous pancreatic carcinoma detected by FDG-PET: two case reports. JOP. 2010;11(1):36–40. 20065550

[11] Ogino T, Ueda J, Sato N, Takahata S, Mizumoto K, Nakamura M, *et al.* Repeated Pancreatectomy for Recurrent Pancreatic Carcinoma after Pylorus-Preserving Pancreatoduodenectomy: Report of Two Patients. Case Rep Gastroenterol. 2010;4(3):429–34. doi:10.1159/000321513. PMC2975012 21060713

[12] Takamatsu S, Ban D, Irie T, Noguchi N, Kudoh A, Nakamura N, *et al.* Resection of a cancer developing in the remnant pancreas after a pancreaticoduodenectomy for pancreas head cancer. J Gastrointest Surg. 2005;9(2):263–9. doi:10.1016/j.gassur.2004.05.016. 15694823

[13] Valle RD, Mancini C, Crafa P, Passalacqua R. Pancreatic carcinoma recurrence in the remnant pancreas after a pancreaticoduodenectomy. JOP. 2006;7(5):473–7. 16998244

[14] Miyazaki M, Yoshitomi H, Shimizu H, Ohtsuka M, Yoshidome H, Furukawa K, *et al.* Repeat pancreatectomy for pancreatic ductal cancer recurrence in the remnant pancreas after initial pancreatectomy: is it worthwhile? Surgery. 2014;155(1):58–66. doi:10.1016/j.surg.2013.06.050. 24238124

[15] Kleeff J, Reiser C, Hinz U, Bachmann J, Debus J, Jaeger D, *et al.* Surgery for Recurrent Pancreatic Ductal Adenocarcinoma. Ann Surg. 2007;245(4):566–72. doi:10.1097/01.sla.0000245845.06772.7d. PMC1877037 17414605

[16] Hashimoto D, Chikamoto A, Ohmuraya M, Sakata K, Miyake K, Kuroki H, *et al.* Pancreatic cancer in the remnant pancreas following primary pancreatic resection. Surg Today. 2014;44(7):1313–20. doi:10.1007/s00595-013-0708-0. 23975591

[17] Lou E, Subramanian S, Steer CJ. Pancreatic cancer: modulation of KRAS, MicroRNAs, and intercellular communication in the setting of tumor heterogeneity. Pancreas. 2013;42(8):1218–26. doi:10.1097/MPA.0000000000000007. 24152947

[18] Ishida J, Toyama H, Matsumoto I, Asari S, Goto T, Terai S, *et al.* Second primary pancreatic ductal carcinoma in the remnant pancreas after pancreatectomy for pancreatic ductal carcinoma: High cumulative incidence rates at 5 years after pancreatectomy. Pancreatology. 2016;16(4):615–20. doi:10.1016/j.pan.2016.05.003. 27237099

[19] Aktualisierte S3-Leitlinie Exokrines Pankreaskarzinom. Zeitschrift für Gastroenterologie 2014; 52(01):146. 10.1055/s-0033-1362168.

[20] National Comprehensive Cancer Network. Clinical Practice Guidelines in Oncology. Pancreatic Adenocarcinoma (Version 2.2017). https://www.nccn.org/store/login/login.aspx?, https://www.nccn.org/professionals/physician_gls/pdf/pancreatic.pdf. Accessed 29 June 2017. 10.6004/jnccn.2017.0131 28784865

